# Sociodemographic determinants associated with parental knowledge of screening services for thalassemia major in Lahore

**DOI:** 10.12669/pjms.35.2.613

**Published:** 2019

**Authors:** Iram Manzoor, Rubeena Zakar

**Affiliations:** 1 *Prof. Iram Manzoor, MBBS, FCPS, MSc, MCPS-HPE, (FCPS). HOD Community Medicine Director Medical Education, Akhtar Saeed Medical and Dental College, Lahore, Pakistan*; 2 *Prof. Rubeena Zakar, MBBS, MPS, PhD. Chairperson and HOD Public Health, Institute of Social and cultural studies, Punjab University Lahore, Pakistan*

**Keywords:** Thalassemia major, Parental awareness, Premarital screening, Prenatal screening

## Abstract

**Objective:**

To assess the sociodemographic factors affecting parental knowledge regarding availability of screening services for thalassemia major.

**Methods:**

A cross sectional study was conducted among biological parents of thalassemic children at three registered centers of blood transfusion in Lahore. A sample of 186 parents was selected through systematic random sampling. A pretested questionnaire was used to collect data. IRB clearance was obtained and informed consent was taken before data collection. Data was analyzed at SPSS version 23 and chi square test was applied and p value was fixed at ≤ 0.05 as significant.

**Results:**

Parents of registered thalassemic children have adequate knowledge about Disease transmission and screening services. 91% of the participants knew that thalassemia major gets transmitted through parents. Cousin marriage was mentioned as a source of transmission by 77% of the participants. 91% of the parents knew about prenatal screening and 89% about premarital screening. Knowledge of parents was significantly associated with male gender, higher education and income.

**Conclusion:**

Male gender, higher education and income are associated with better knowledge of parents.

## INTRODUCTION

Thalassemia is one of the most common inherited blood disorders in the world.^1^ Worldwide 240 million heterozygous and 200,000 homozygous thalassemic children are born annually.^2^ It is estimated that about 3-5% of the world’s population carries gene for beta-thalassemia trait.^3^ Highest frequency of carrier for β thalassemia is reported in Southeast Asia.^4^ According to WHO thalassemia is spreading steadily with varying prevalence rate in different regions. This rate varies from 1% in Thailand, 2% in Malaysia, 4% in Jordan and 17% in India.^5^ In Pakistan over 5000 children with thalassemia major are born every year. Pakistan carries a risk of 6% of the population as heterozygous carriers serving as potential source of spread of thalassemia major.^6^

This high rate of transmission of thalassemia through healthy, undiagnosed carriers can only be controlled through health education and through community screening.^7^ Worldwide genetic counseling, premarital screening and prenatal screening has shown great impact in reduction of incidence of thalassemia major.^8^ In Pakistan, only Punjab thalassemia prevention program is targeting genetic counseling, premarital and prenatal screening services for thalassemia major. No significant work is observed in terms of establishment of prevention program is seen in other provinces of Pakistan. Despite this program in Punjab, a recent study in Rawalpindi showed that 52.2% of the parents of thalassemic children were not counseled for the modes of transmission and prevention of diseases.^9^ A recent study conducted in India showed that genetic etiology was known to only 47.6% of the caregivers of thalassemic children. It was also assessed that knowledge about screening services ranged between 50-52%.^10^ Many factors affect the knowledge of transmission of disease and option of preventive services. A study conducted in Thailand showed that lack of maternal awareness related to antenatal screening is related to age, education, multi-gravidity and family history of thalassemia.^11^ A study conducted in Pakistan assessed that only 33% of the parents of thalassemic children had knowledge about premarital screening for detection of thalassemia trait and 76.5% had knowledge of prenatal screening.^12^ There is lack of scientific evidence on factors affecting parental knowledge about thalassemia major in Pakistan. The objective of this study is to find out the sociodemographic factors which affect parental knowledge about disease transmission and provision of screening services for thalassemia major available in Pakistan.

## METHODS

A cross sectional survey was conducted between January to May 2017 in three registered centers for blood transfusion for children suffering from thalassemia major. These centers included one government owned, Sir Ganga Ram Hospital, Lahore and two private owned, Fatimid foundation and Sundas Foundation. The respondents used to collect data were biological parents, accompanying their children for blood transfusions to theses registered centers. The registered population of thalassemic children in these referral centers was less than 5000. On the basis of assumption of known population with prevalence rate of 6%, WHO online calculator was used to assess the sample size of 186 parents (father/ mother) to be included in the sample. A probability type of Systematic random sampling technique was used to collect sample in registered patients of thalassemia major. All other patients coming for blood transfusion of other hematological disorders (hemophilia) were excluded from the sample. A pretested questionnaire was used to collect data containing multiple sociodemographic variables, related to knowledge of disease transmission and screening services were included in the tool. Data was collected anonymously keeping all the information confidential. Data was collected in separate rooms with detailed, structured interviewing technique. Ethical review board clearance was obtained from Punjab University and detailed tools were provided to all places of data collection before seeking approval to collect data. Research was conducted abiding by all principles laid down in Helsinki’s declaration keeping in view all physical and emotional needs of the participants. Data was analyzed through SPSS version 23 and descriptive presentation was given in forms of tables, graphs and charts. Chi square test of significance was applied to assess association between socio-demographic variables and knowledge levels. Significant association was declared at ≤0.05 and presented in tables.

## RESULTS

In this cross sectional survey, 186 participants were included which were uniformly distributed in three registered blood transfusion centers of Lahore. From Each registered center, 62 (33.3%) participants was taken, including Sir Ganga Ram Hospital Lahore, Sundas foundation and Fatimid foundation. In this study, Out of 186 total participants, 132 (71.0%) were mothers and 54 (29.0%) were fathers who participated in the study. Majority of the participants belonged to ages group between 30-40 years comprising 77(41.4%) of the participants. Patient aged between 20-30 years had 52(28.0%) participants. Educational status of the parents showed a major proportion 53 (28.5%) belonging to illiterate group. A vast majority of mothers, 117 (62.9%) were house wives. A small fraction of fathers 6 (0.03%) were unemployed too. Out of 186 participants, 109 (58.6%) belonged to a group where monthly income was below Rs 20,000 (Table-I).

**Table-I:** Socio demographic profile of respondents: (n=186).

Socio demographic variables	Frequency (n)	Percentage (%)
***Age Distribution***		
20- 30 years	52	28.0
30-40 years	77	41.4
40-50 years	42	22.6
50-60 years	15	8.1
***Gender Distribution***		
Mothers	132	71.0
Fathers	54	29.0
***Educational status***		
Illiterate	53	28.5
Primary pass	21	11.3
Middle Pass	30	16.1
Matric pass	42	22.6
Inter pass	14	7.5
Graduate	16	8.6
Masters	10	5.4
***Employment status***		
House wife mothers	117	62.9
Working mothers	15	0.08
Working fathers	48	25.8
Unemployed fathers	6	0.03
***Monthly Income***		
Less than 20,000	109	58.6
20 to 30,000	37	19.9
30-40,000	14	7.5
40-50,000	11	5.9
50,000 above	15	8.1

**Fig.1:**
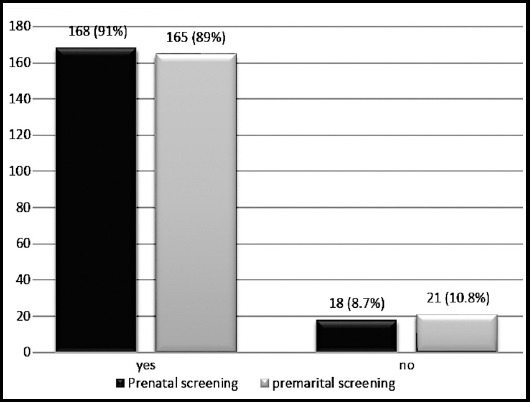
Awareness about provision of screening services.

Out of 186 participants in this study, 169 (90.9%) were aware about the genetic transmission of disease. One hundred and forty three participants (76.9%) were aware that cousin marriages play a role in transmission of thalassemia major while 39 (21%) were ignorant of this fact. A significant number of 168 (91%) of the respondents were aware of the fact that Thalassemia major can be detected in affected child during pregnancy while 18 (8.7%) were not aware about prenatal screening (Fig.1).

One hundred and sixty five respondents which constituted 89.2% were aware of the fact that thalassemia trait can be detected before marriage. It was assessed that there was a great proportion of participants, 164 (88.6%) who mentioned that they had been counseled regarding disease transmission and its preventive strategies by the counseling staff of the registered centers which included doctors, nurses and other staff members.(Table-II).

**Table-II:** Parental awareness about transmission of Thalassemia major.

Socio demographic variables	Frequency (n) n=186	Percentage (%)
Awareness about transmission of disease through parents (Genetic Transmission)		
Yes	169	90.9
No	11	5.9
Don’t know	6	3.2
Awareness about transmission of disease through cousin marriage		
Yes	143	76.9
No	39	21.0
Don’t know	4	2.2
Awareness about detection of Thalassemia Major during pregnancy		
Yes	168	91.3
No	18	8.7
Awareness about detection of Thalassemia trait before marriage		
Yes	165	89.2
No	21	10.8

Sociodemographic factors were cross tabulated with knowledge of parents related to provision of prenatal and premarital screening services availability. It was observed that no significant association was found between age group of participants and their knowledge about screening services. There was significant difference in the knowledge of two genders regarding provision of premarital screening. Females had less knowledge as compared to males regarding detection of thalassemia trait in premarital phase (p= 0.038). Educational status had significant association with knowledge about provision of screening services. People were unaware about screening in the group which was less educated for prenatal screening (p = 0.025) and for premarital screening (p = 0.017) as well. Housewives (mothers which were not working) had poor knowledge about both types of screening services with p values of 0.002 and 0.007 respectively. Significant association was seen in lack of awareness and lower income group with p values of 0.002 in both groups where Income group of less than Rs. 30,000 per month had poor knowledge as compared to the group having income more than Rs.30,000 per month (Table-III)

**Table-III:** Cross tabulation of socio demographic profile with awareness about provision of prenatal and premarital screening services.

Sociodemographic Profile	Knowledge about prenatal screening		p value	Knowledge about premarital screening		p value

Age distribution	Yes n=168	No n=18		Yes n=165	No n=21	
20-30 years	47	4		46	6	
30-40 years	71	6	0.394	69	8	0.693
40-50 years	38	4		38	4	
50-60 years	12	4		12	3	
***Gender distribution***						
Males	47	8		44	4	
Females	121	10	0.185	121	17	0.038*
***Educational status***						
Illiterate	45	9		44	10	
Primary	18	3		17	4	
Middle	29	1		27	2	
Matric	36	5	0.025*	37	5	0.017*
Intermediate	14	0		14	0	
Graduate	16	0		16	0	
Masters	10	0		10	0	
***Employment status***						
Housewife mothers	100	17		98	20	
Working mothers	15	0		15	0	
Unemployed fathers	4	1	0.002*	4	1	0.007*
Working fathers	48	0		48	0	
***Income status***						
< Rs.20,000	94	13		94	14	
Rs.20,000- 30,000	34	5		31	6	
Rs.30,000- 40,000	14	0	0.002*	14	0	0.002*
Rs.40,000- 50,000	11	0		11	0	
>Rs.50,000	15	0		15	0	

## DISCUSSION

Thalassemia major ranks in the top category of genetically transmitted blood disorder in Pakistan. In Pakistan the estimated carrier rate for beta thalassemia is 5-7% which results in 4000- 7000 affected births annually.^13^ Despite of the alarming increase in annual births of children with thalassemia major, no intervention has been planned at national level. Punjab and Sindh thalassemia prevention programs offers genetic counseling, prenatal and premarital screening for the control of thalassemia major at provincial levels.^14^ These programs also target to increase awareness about disease transmission and provision of screening services. Multiple studies were conducted to assess knowledge of parents and care providers of thalassemic children about availability of screening services. A study in Lahore concluded that 76.5% of the parents have knowledge about prenatal screening but only 33% of the parents knew that thalassemia trait can be detected before marriage.^15^

Many sociodemographic factors affect the knowledge of parents regarding disease transmission and its prevention. This study targeted to find out those factors which affect parental knowledge. In this study it was observed that 71% of the mothers and 29% of the fathers accompanied their children for transfusion services. A study conducted in Taiwan assessed that Scores of maternal knowledge which were high and were positively correlated with the scores of knowledge of thalassemic children too.^16^ The results of this study concluded that no significant difference was observed in terms of knowledge difference between fathers and mothers for the provision of prenatal screening services but fathers were more aware regarding provision of premarital screening. A study was conducted in care givers of thalassemic children which assessed that fathers had better understanding of disease in their children and had exhibited better coping strategies as compared to mothers.^17^ Another study was conducted in India which indicated poor knowledge of parents irrespective of their gender regarding needs of thalassemic children and further preventive strategies.^18^ The results of this study showed that no significant association was found in different age group of parents and their awareness level about screening tests. Similar results were observed in a study conducted at India where parental awareness level was compared at Government owned facility and Nongovernment based organization.^19^

Results of this study showed significant association in the lower educational status and poor knowledge about availability of screening tests and same results have been formulated by study conducted in Karachi in 2008.^20^ Another study conducted to evaluate children with βthalassemia to assess their self-concept, behavior and parental attitudes. It was assessed that parental education level was significantly associated with knowledge of parents regarding disease transmission and associated risks.^21^ The results of another study conducted in China showed that minorities had very little knowledge about thalassemia major.^22^ In Iran Thalassemia prevention program established counseling and screening at primary care facility. One of the major success of this program is creating awareness in general population at national level irrespective of age, gender and socioeconomic group. A study conducted in Malaysia also revealed that mean knowledge score was significantly associated with higher age group, higher education attainment, employment status and high household income groups.^23^ The results were consistent with the results of this study except that no association was found in age group and knowledge level in this study. A study conducted in Saudi Arabia in 2008 showed that those couples who received counseling had better knowledge about thalassemia major as compared to general population irrespective of their age and gender.^24^ Targeting the high risk group with counseling for prenatal and premarital screening can result in 92% reduction in births of children affected with thalassemia major.^25^

## CONCLUSION

The study concludes that significant association was found with male gender, higher education, high income and employment status of the parents with adequate knowledge about disease transmission and provision of screening tests.

## RECOMMENDATIONS

This study recommends that health education programs for prevention of thalassemia should target general population and population at risk irrespective of their age, gender, education and employment status. The need of the hour is to create mass awareness for prevention of this important public health disease.

### Authors’ Contributions

**IM:** Conceptualization, Literature search, Write up.

**RZ:** Review of methodology, Designing of questionnaire, Supervision of write up.
